# Overexpression of a Defensin-Like Gene *CAL2* Enhances Cadmium Accumulation in Plants

**DOI:** 10.3389/fpls.2020.00217

**Published:** 2020-02-27

**Authors:** Jin-Song Luo, Yan Xiao, Junyue Yao, Zhimin Wu, Yong Yang, Abdelbagi M. Ismail, Zhenhua Zhang

**Affiliations:** ^1^Southern Regional Collaborative Innovation Center for Grain and Oil Crops in China, College of Resources and Environmental Sciences, Hunan Agricultural University, Changsha, China; ^2^Hunan Provincial Key Laboratory of Farmland Pollution Control and Agricultural Resources Use, Hunan Provincial Key Laboratory of Nutrition in Common University, National Engineering Laboratory on Soil and Fertilizer Resources Efficient Utilization, Changsha, China; ^3^International Rice Research Institute, Metro Manila, Philippines

**Keywords:** plant defensin, *CAL2*, cadmium accumulation, *Arabidopsis*, rice

## Abstract

Accumulation and detoxification of cadmium in rice shoots are of great importance for adaptation to grow in cadmium contaminated soils and for limiting the transport of Cd to grains. However, the molecular mechanisms behind the processes involved in this regulation remain largely unknown. Defensin proteins play important roles in heavy metal tolerance and accumulation in plants. In rice, the cell wall-localized defensin protein (CAL1) is involved in Cd efflux and partitioning to the shoots. In the present study, we functionally characterized the CAL2 defensin protein and determined its contribution to Cd accumulation. CAL2 shared 66% similarity with CAL1, and its mRNA accumulation is mainly observed in roots and is unaffected by Cd stress, but its transcription level was lower than that of *CAL1* based on the relative expression of *CAL2*/*Actin1* observed in this study and that reported previously. A promoter-*GUS* assay revealed that *CAL2* is expressed in root tips. Stable expression of the *CAL2-mRFP* fusion protein indicated that *CAL2* is also localized in the cell walls. An *in vitro* Cd binding experiment revealed that CAL2 has Cd chelation activity. Overexpression of *CAL2* increased Cd accumulation in *Arabidopsis* and rice shoots, but it had no effect on the accumulation of other essential elements. Heterologous expression of *CAL2* enhanced Cd sensitivity in *Arabidopsis*, whereas overexpression of *CAL2* had no effect on Cd tolerance in rice. These findings indicate that CAL2 positively regulates Cd accumulation in ectopic overexpression lines of *Arabidopsis* and rice. We have identified a new gene regulating Cd accumulation in rice grain, which would provide a new genetic resource for molecular breeding.

## Introduction

Excessive accumulation of cadmium (Cd), a heavy metal, can cause damage to different species (Clemens et al., 2013). China’s rapid industrialization in the past three decades has led to increasing contamination of agricultural soils with Cd, and its content in the grains of rice grown in parts of southern China has been reported to exceed Chinese safety standards. This has led to widespread concern about food safety (Wang et al., 2019). Exposure to elevated levels of Cd affects the homeostasis of essential elements, destroys the photosynthetic system, causes oxidative stress, and inhibits plant growth and development (Moulis, 2010). Excessive accumulation of Cd in the body can cause Itai-Itai disease in humans (Kazantzis, 2004). Phytoremediation combined with lower Cd content in grains could effectively address Cd pollution and its associated health hazards, and necessities comprehensive understanding of the mechanisms involved in Cd accumulation and detoxification in plants.

Numerous studies had reported on the mechanisms for detoxification and accumulation of Cd in plants. In brief, cytoplasmic chelation and vacuolar sequestration are the main detoxification pathways for Cd in plants and yeast (Song et al., 2003, 2010; Hanikenne et al., 2008; Verbruggen et al., 2009; Ueno et al., 2010; Chao et al., 2012; Park et al., 2012; Huang et al., 2012). Cd accumulation in plant tissues, its uptake, and redistribution through long-distance transport are mainly mediated via transporters of essential elements (Morel et al., 2008; Korenkov et al., 2009; Wong and Cobbett, 2009; Nocito et al., 2011; Satoh-Nagasawa et al., 2011; Sasaki et al., 2012; Takahashi et al., 2012; Yang et al., 2014; Yan et al., 2016).

Another class of molecules that may be involved in Cd accumulation and detoxification are plant defensins, a large family of highly conserved and stable proteins that are considered natural immune molecules with a wide range of biological functions (Parisi et al., 2019). These peptides have a spherical three-dimensional structure stabilized by four disulfide bonds and share a CSαβ motif composed of an α-helix and a triple-stranded anti-parallel β-sheet (Parisi et al., 2019). Research over the past few decades revealed several functions of these molecules; for example, human defensin 5 (HD5) have Zn/Cd binding activity (Zhang et al., 2013). Other studies have shown that plant defensin gene type 1 (PDF1s) increases tolerance to zinc in yeast and plants (Mirouze et al., 2006; Shahzad et al., 2013; Nguyen et al., 2014; Mith et al., 2015). In addition, Cd stress was reported to significantly induce the expression of *PDF1.2* in *Arabidopsis*, whereas Zn supply decreased its expression in a Zn/Cd hyperaccumulator, *Noccaea caerulescens* (Cabot et al., 2013; Gallego et al., 2016).

The *Arabidopsis* defensin type 2 family (PDF2s) has six members (Thomma et al., 2002). Electrophysiological studies have shown that AtPDF2.3 has potassium channel blocking activity (Kim et al., 2016). AtPDF2.5 is a Cd-induced protein that is located in cell walls of the vascular bundles in the xylem; it positively regulates Cd tolerance and accumulation by mediating the chelation and efflux of cytoplasmic Cd to the apoplast cell wall in *Arabidopsis thaliana* (Luo et al., 2019b). *AtPDF2.6* is strongly induced by Cd stress and is located in the cytoplasm of xylem vascular bundles. By chelating cytoplasmic Cd, AtPDF2.6 can enhance the tolerance of *Arabidopsis* to Cd. These findings challenge the generally accepted view that defensins are secreted proteins (Luo et al., 2019a). Recent xylem sap proteomic studies revealed that plant defensins play a positive role in the Cd tolerance of *Brassica napus* (Luo and Zhang, 2019).

Sequencing and annotation studies identified thousands of plant defensins in rice (Kawahara et al., 2013). The quantitative trait locus of Cd accumulation in rice leaf 1 (CAL1) was map-based cloned and was found to encode a defensin-like protein (Luo et al., 2018). CAL1 was functionally characterized as a positive regulator of Cd accumulation in rice leaves (Luo et al., 2018). CAL1 is expressed preferentially in the root exodermises and xylem parenchyma cells. It is also involved in Cd chelation and secretion from the cytosol to extracellular spaces and can consequently lower the cytosolic Cd concentration and drive long-distance Cd transport via the xylem vessels (Luo et al., 2018). In this study, we functionally characterized a cell wall-localized defensin, CAL2, and determined its contribution to Cd accumulation. CAL2 was found to be the closest homolog of CAL1, and its mRNA accumulation was mainly detected in root tips and was unaffected by Cd stress. Our findings indicate that CAL2 positively regulates Cd accumulation in ectopic overexpression lines of rice and *Arabidopsis*, and provides a new genetic resource for molecular breeding.

## Materials and Methods

### Sequence Analysis and Construction of Phylogenetic Tree

*Arabidopsis* and rice defensin protein sequences were downloaded from the TAIR database and RAP-DB, respectively. Sequence alignment was performed using ClustalW. The phylogenetic tree was constructed by MEGA 5 software after ClustalW alignment.

### Plant Material, Growth Conditions, and Elemental Analysis

We used the wild-type rice (*Oryza sativa* L.) cultivar, ZH11. Plants were grown in paddy fields contaminated with heavy metals and seeds were harvested at maturity. Alternatively, seeds were pretreated and uniformly germinated seeds were planted in 96-well plates with their bottoms removed. Rice seedlings were grown hydroponically in Yoshida solution (pH 5.8), as previously described (Yoshida et al., 1976), at 28°C and 60% relative humidity, with a 13-h light/11-h dark photoperiod as described earlier (Luo et al., 2018). For the Cd tolerance assay, rice seedlings were grown hydroponically for 14 days in the presence of 0 or 5 μM Cd, and then sampled to determine the fresh weights of shoot and roots. The Cd concentration in leaf, xylem sap, and root was determined by inductively coupled plasma mass spectrometry (ICP-MS, ELAN DRC-e, PerkinElmer), as previously described (Gong et al., 2003). Xylem sap was extracted and assayed as described by Luo et al. (2018).

Seeds of the wild-type *A. thaliana* (Col-0) and *CAL2* heterologous overexpression lines were surface sterilized, germinated on half-strength Murashige and Skoog (1/2 × MS) medium, containing 0.8% (w/v) sucrose, 0.1% (w/v) 2-(*N*-morpholino)ethanesulfonic acid (MES), and 0.8% (w/v) agar; and kept at 22°C under 300 μmol m^–2^ s^–1^ photosynthetically active radiation, and a 16-h-light/8-h-dark cycle for 7 days. Seedlings were then transferred to a quarter-strength hydroponic solution, as described previously (Arteca and Arteca, 2000; Gong et al., 2003; Luo et al., 2019b). When the plants are 4 weeks old, they were exposed to 10 μM CdCl_2_ for 3 days, as described by Luo et al. (2019a), after which the roots and shoots were harvested. The concentration of Cd in plant tissues was determined by ICP-MS (Gong et al., 2003). Cd tolerance and nitrate concentration were determined in 4 weeks-old plants, subjected to 20 μM CdCl_2_ for 3 days before tissue sampling. Nitrate was extracted and assayed as described in Li et al. (2010).

### DNA Constructs and Transformation of Plants

To clone the *CAL2* promoter, total DNA was extracted from ‘ZH11’ with TPS buffer (100 mM Tris-HCL pH 8.0; 10 mM EDTA pH 8.0; 1 M KCl). A 1490-bp genomic fragment immediately upstream of the *CAL2* start codon was PCR-amplified using the primers, ProCAL2F and ProCAL2R (Supplementary Table S1). The resulting ProCAL2 promoter fragment was then sub-cloned into the binary vector pCAMBIA1300-GUS using a one-step cloning kit (C112-01, Vazyme Biotech Co., Ltd.). The pCAMBIA1300-GUS vector was recovered after *Hin*dIII/*Bam*HI restriction digestion. To determine the subcellular localization of CAL2 in *A. thaliana*, the *35S*:*mRFP* fragment was recovered from *35S*:*mRFP*/PA7 by *Hin*dIII/*Sac*I restriction digestion (Luo et al., 2018), and the resulting *35S*:*mRFP* fragment was inserted into pCAMBIA1300 to generate the *35S*:*mRFP*/pCAMBIA1300 construct. The coding sequence of *CAL2*, without a stop codon, was PCR-amplified using the primers CAL2F and CAL2R (Supplementary Table S1). The resulting fragments were fused in-frame to the 5′-terminus of the monomer red fluorescent protein gene (*mRFP*) to generate the *35S*:*CAL2-mRFP*/pCAMBIA1300 construct, using the one-step cloning kit (C112-01, Vazyme Biotech Co., Ltd). All resulting constructs were transformed into *A. thaliana* using the floral dip method (Clough and Bent, 1998). Transgenic plants were screened using hygromycin B and confirmed by quantitative PCR. Alternatively, the *35S*:*CAL2-mRFP*/pCAMBIA1300 construct was transformed into ZH11 at Wuhan Bo Yuan Biotech. Co. Ltd, as previously described (Hiei et al., 1994).

### Expression Assay

Rice seedlings were grown in hydroponic solution for 2 weeks before the treatments and then sampled for further analysis. Alternatively, tissues were sampled from rice plants grown in paddy fields at the heading stage. Total RNA was extracted from the collected tissues using TRIzol reagent according to the manufacturer’s instructions (Invitrogen). Complementary DNA was synthesized using the PrimeScript^TM^ RT Kit with gDNA Eraser (Perfect Real Time; TAKARA) following the manufacturer’s protocol. The relative expression of target genes was determined by quantitative reverse transcription polymerase chain reaction (RT-qPCR), performed on an Applied Biosystems StepOne^TM^ Real-Time PCR System with SYBR Premix Ex-Taq (TAKARA), according to the manufacturer’s instructions. The primers used in these assays are listed in Supplementary Table S1.

### Histochemical and Subcellular Localization Analyses

To confirm the histological expression pattern of β-glucuronidase (GUS) driven by the proCAL2 promoter, histochemical staining was performed using a GUS histochemical assay kit (Real-Times, China) following the manufacturer’s protocol. The seedlings were grown in 1/2 MS medium for 1 week. The roots of the transgenic lines were subjected to mRFP imaging using confocal microscopy (Zeiss; LSM880).

### Protein Purification and Related Assays

Fragments of Δ*SPCAL2* (a truncated form of *CAL2* representing the mature CAL2 protein of 33–82 amino acids, which lacks the signal peptide sequence that spans amino acids 1–32) were PCR-amplified using the primers TF-ΔSPCAL2F and TF-ΔSPCAL2R (Supplementary Table S1) and cloned into the pCold-TF vector. Expression of the recombinant proteins was induced by 0.2 mM isopropyl β-D-1-thiogalactopyranoside at 16°C for 10 h. The cells were further grown for 10 h in the presence of 100 μM CdCl_2_ before collection, and then lysed using BugBuster Master Mix (Novagen), as previously described (Luo et al., 2018). The cell debris was removed by centrifugation at 16000 × *g* for 15 min. The supernatant was purified using Ni-NTA agarose (Qiagen), and free Cd was removed using Zeba Spin Desalting Columns (Thermo Scientific). Five micrograms of purified protein per sample was loaded onto 12% SDS-PAGE gels and detected using Coomassie blue staining. Both procedures were performed at Sangon Biotech Co., Ltd. The purified proteins were divided into two portions: one was used to measure the Cd content, using ICP-MS, and the other to measure the protein concentration (Bio-Rad, DC Protein Assay Kit). The metal-to-protein stoichiometry was calculated based on Cd and protein concentrations, as described previously (Wan and Freisinger, 2009).

### Statistical Analyses

All experiments were conducted using a completely randomized design. Three independent biological samples were used as replicates and two technical replicates were used for each treatment. Data were analyzed using two-tailed Student’s *t*-tests and differences were deemed significant at *P* < 0.05 and highly significant at *P* < 0.01.

### Accession Numbers

Sequence data from this study can be found in RAP-DB under the following accession number: *CAL2* (*Os04g0522100*). Additional sequence data are available in Supplementary Table S1.

## Results

### CAL2 Is the Closest Homolog of CAL1

**FIGURE 1:**
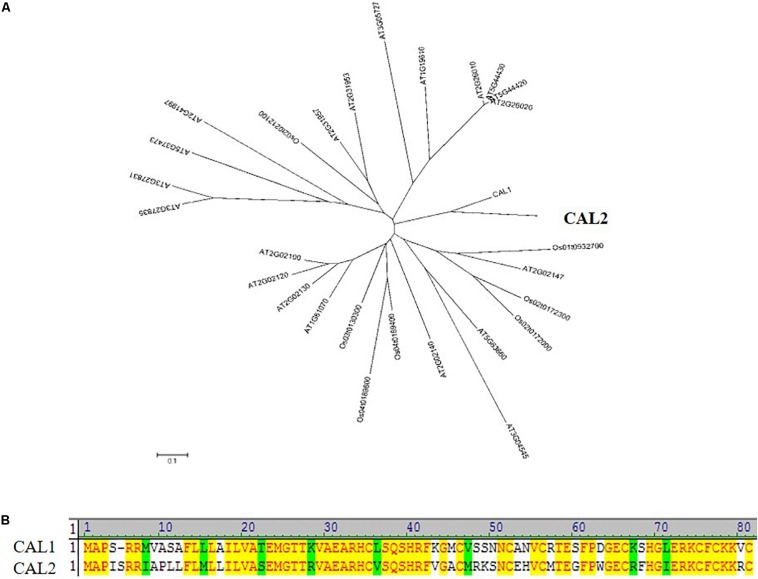
CAL2 is the closest homolog of CAL1. **(A)** Phylogenetic relationship of defensin proteins in rice and *Arabidopsis thaliana*. The 0.1 scale shows substitution distance. **(B)** Alignment of CAL1 and CAL2 proteins.

CAL2 showed a high degree of similarity to CAL1 (Figure 1A), with 66% of the sequences being identical (Figure 1B). The CAL2 sequence comprised 82 amino acid residues (9.4 kDa). Bioinformatics analysis indicated that CAL2 contains an N-terminal signal peptide and a C-terminal cysteine-rich domain. The signal peptide cleavage site is between amino acid residues 32 and 33 (Supplementary Figure S1).

### *CAL2* Is Mainly Expressed in Roots and Does Not Respond to Cd Stress at the mRNA Level

**FIGURE 2:**
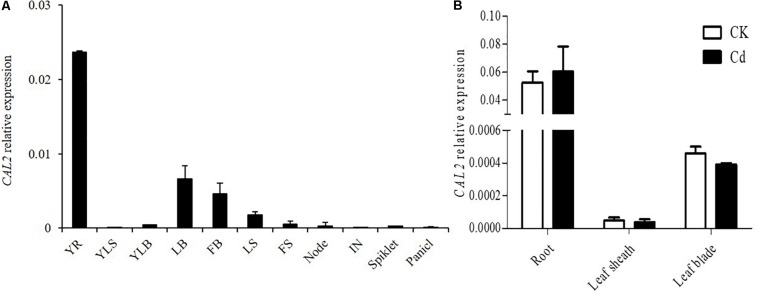
Expression pattern of *CAL2*. **(A)** *CAL2* expression in various rice tissues. YR, young root; YLS, young leaf sheath; YLB, young leaf blade; LB, leaf blade; FB, flag leaf blade; LS, leaf sheath; FS, flag leaf sheath; IN, internode. **(B)** *CAL2* expression in roots of 2-week-old seedlings treated with Cd at 0 or 100 μM Cd for 6 h. Data are means ± standard deviations of three independent biological replicates and normalized to *Actin1*.

The expression of *CAL2* was investigated in different tissues at the seeding and flowering stages. Quantitative RT-PCR results showed that *CAL2* was mainly expressed in the roots (Figure 2A). To investigate whether the expression of *CAL2* is affected by Cd stress, we exposed the rice seedlings to solutions containing 0 or 100 μM of Cd. The expression of *CAL2* was not significantly changed in roots, leaf sheaths, and leaf blades exposed to either solution (Figure 2B).

### Tissue Specific Expression and Subcellular Localization of *CAL2*

**FIGURE 3:**
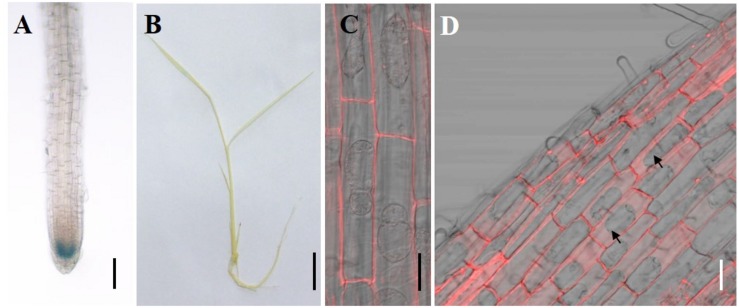
Tissue specificity expression and subcellular localization assay. **(A,B)** Histochemical assay of *CAL2* promoter GUS activity in 14-day-old rice seedlings. **(C,D)** Subcellular localization of CAL2 in *Arabidopsis* **(C)** and rice **(D)** plants harboring the construct *35S:CAL2-mRFP*. 7-day-old seedling root epidermal cells were incubated in 40% sucrose to induce plasmolysis and then imaged by confocal microscopy. The arrows indicate the plasmolyzed cells. Scale bars = 2 mm in **(A)**, 1 cm in **(B)**, and 20 μm in **(C,D)**. Two independent transgenic lines were used in **(A–D)** and showed similar results.

To investigate the tissue specificity of *CAL2* expression, we determined the expression pattern of *CAL2* by histochemical analysis of GUS expression driven by the *CAL2* promoter. Consistent with the quantitative RT-PCR results, GUS activity was mainly detected in the root tips (Figure 3A), whereas it was not detected in the leaf sheaths and leaf blades (Figure 3B). To determine subcellular localization, we transformed *CAL2*:*mRFP* into *Arabidopsis* and rice under the control of the *35S* promoter. The fluorescence produced by the mRFP fusion protein was detected in the cell wall (Figures 3C,D).

### CAL2 Has Cd Binding Activity

**FIGURE 4:**
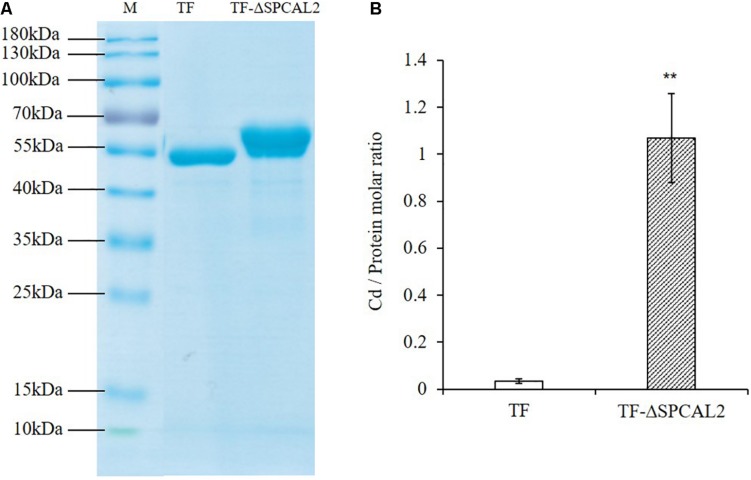
*In vitro* Cd binding assay. **(A)** *Escherichia coli* trigger factor (TF) and TF fused to the N terminus of the signal peptide-deleted CAL2 (TF-ΔSPCAL2) purified from *E. coli*. **(B)** The molar ratio of Cd against TF-ΔSPCAL2 protein purified from *E. coli* cells grown with 100 μM CdCl_2_. Data are mean ± standard deviations of three independent biological replicates. Significant differences were determined by Student’s *t*-test (**P < 0.01).

*CAL2* encodes a small cysteine-rich peptide that contains eight cysteine residues at the C-terminus and may have Cd chelating activity. To test this hypothesis, we expressed and purified recombinant TF-CAL2 proteins in *Escherichia coli* cells treated with Cd. TF is a prokaryotic ribosomal binding chaperone protein that facilitates the co-translation and folding of newly synthesized peptide chains. Compared with tags from other biological sources, TF has higher expression efficiency and better solubility in *E. coli* (Luo et al., 2019a). Coomassie blue staining results indicated that we successfully purified TF; and TF fused to the N-terminus of the signal peptide-deleted CAL2 (TF-ΔSPCAL2) proteins from *E. coli* transformants treated with 100 μM CdCl2 (Figure 4A). The ICP-MS analysis showed that TF-ΔSPCAL2 has Cd chelating activity; the binding molar ratio of TF-ΔSPCAL2 to Cd is 1 (Figure 4B).

### Heterologous Expression of *CAL2* Increases Cd Allocation to Shoot in *Arabidopsis*

**FIGURE 5:**
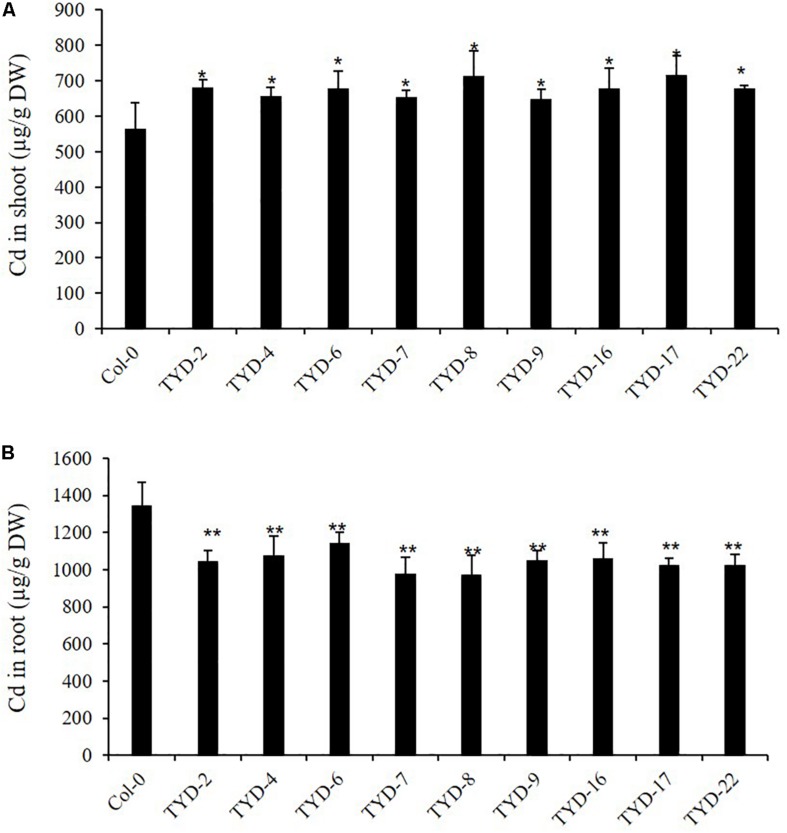
Ectopic expression of *CAL2* increased Cd allocation to shoot in *Arabidopsis*. Four weeks old *35S:CAL2-mRFP* transgenic plants were exposed to 10 μM Cd for 3 days. **(A)** Heterologous overexpression of *CAL2* increased Cd concentration in Arabidopsis shoots. **(B)** Heterologous overexpression of *CAL2* decreased Cd concentration in Arabidopsis roots. Data are mean ± standard deviations of three independent biological replicates. Significant differences from Col-0 were determined by Student’s *t*-test (*P < 0.05, **P < 0.01).

Next, we studied the effect of heterologous overexpression of *CAL2* on metal transport and distribution in *A. thaliana*. We first detected the transcription level of *CAL2* in the homozygously overexpressing *A. thaliana* lines and found that the expression level of *CAL2* was significantly higher than that in the wild-type Col-0 plants (Supplementary Figure S2A). Four-week old hydroponically grown *35S*:*CAL2-mRFP* transgenic and wild-type Col-0 plants were exposed to 10 μM Cd for 3 days. Compared with that in the wild-type Col-0, the heterologous overexpression of *CAL2* significantly increased the accumulation of Cd in *Arabidopsis* shoots (Figure 5A), while the concentration of Cd in roots decreased significantly (Figure 5B). There was no difference in the concentration of other essential metals between the wild-type Col-0 and *Arabidopsis* plants heterologously overexpressing *CAL2* (Supplementary Figure S2B). These data indicate that CAL2 may positively regulate Cd allocation in *Arabidopsis*.

We selected representative transgenic lines expressing *CAL2* for the Cd tolerance assay. When hydroponically grown without Cd, transgenic lines TYD-6 and TYD-7 showed similar growth as the wild-type Col-0 (Supplementary Figure S3A). However, when 4-week-old hydroponically grown *35S*:*CAL2-mRFP* transgenic and wild-type Col-0 plants were exposed to 20 μM Cd for 3 days, the TYD-6 and TYD-7 lines showed greater Cd sensitivity than the wild-type control plants (Supplementary Figure S3A). Previous research revealed that Cd/Na stress influences nitrate allocation to the roots, which is regulated by the nitrate transporters, NRT1.8 and NRT1.5, and functions in promoting stress tolerance (Li et al., 2010; Zhang et al., 2014, 2018). To investigate the potential mechanisms of cadmium sensitivity, nitrate concentrations in *A. thaliana* shoots and roots were measured and normalized to fresh weight under different growth conditions. Under control conditions, the root/shoot nitrate ratio was similar in Col-0, TYD-6, and TYD-7 plants (Supplementary Figure S3B). However, when plants were exposed to 20 μM Cd for 3 days, the root/shoot nitrate ratio significantly decreased in the TYD-6 and TYD-7 plants, compared to that in the wild-type Col-0 plants (Supplementary Figure S3B). These results indicate that CAL2 may negatively regulate the root/shoot nitrate ratio under Cd stress conditions to decrease Cd tolerance in *Arabidopsis*.

### Overexpression of *CAL2* Increases Cd Accumulation in Rice

**FIGURE 6:**
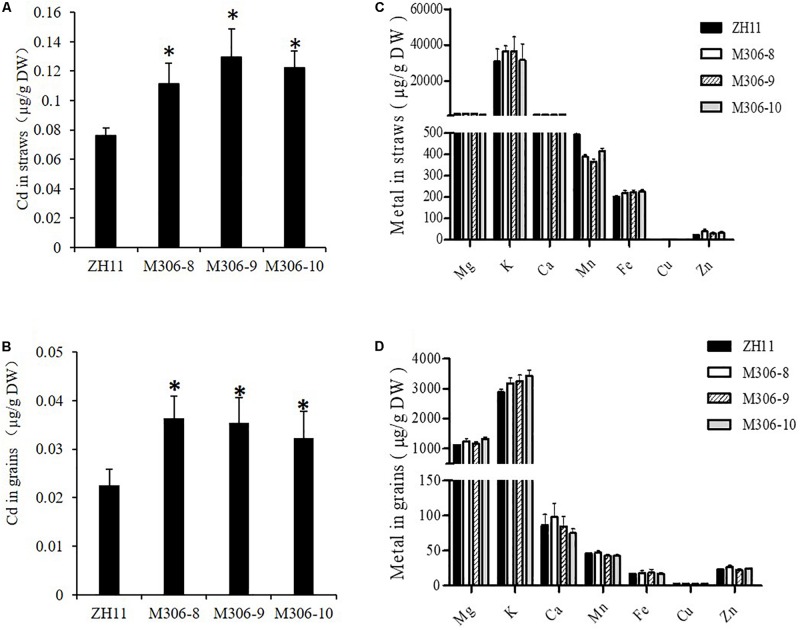
Overexpression of *CAL2* increased Cd accumulation in rice. Transgene-negative control ZH11 and *35S:CAL2-mRFP* transgenic plants were grown in a heavy metal contaminated paddy field until ripening. Heterologous overexpression of CAL2 increased cadmium (Cd) accumulation in rice straws **(A)** and grains **(B)**. **(C,D)** Essential mineral nutrients accumulation in **(C)** rice straws and **(D)** grains. Data are means ± SDs of three independent biological replicates. Significant differences from ZH11 were determined by Student’s *t*-test (^∗^*P* < 0.05).

To study the role of CAL2 in rice, we overexpressed it in a representative rice cultivar (ZH11) under the control of the *35S* promoter and selected three representative overexpression lines for further experiments (Supplementary Figure S4). Compared with the transgenic negative control ZH11, the Cd concentration in straw and seeds significantly increased in the *CAL2*-overexpression lines, when grown in a Cd-contaminated field (Figures 6A,B). There was no difference in the concentration of other essential metals in the straw and seeds between the transgenic negative control ZH11 and *CAL2*-overexpression lines (Figures 6C,D).

**FIGURE 7:**
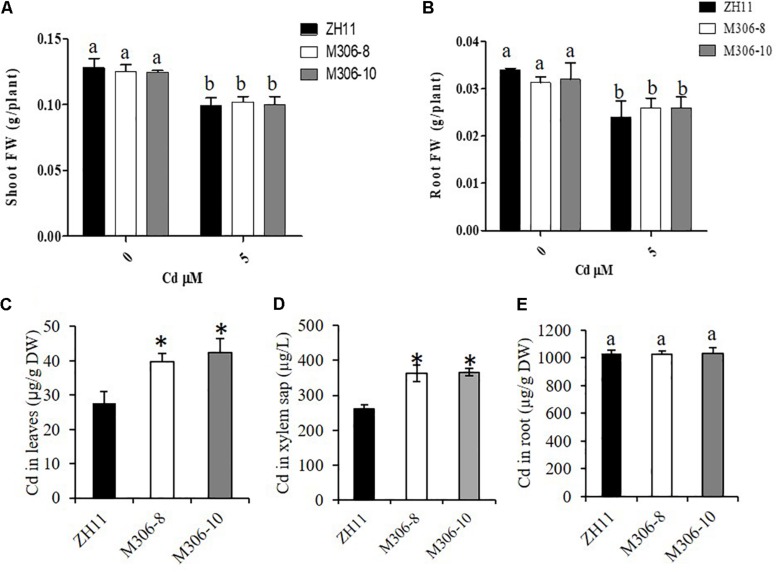
Cd tolerance assays of *CAL2* overexpression lines. Transgene-negative control ZH11 and *35S:CAL2-mRFP* transgenic rice seedlings were grown for 14 days in hydroponics supplemented with 0 or 5 μM Cd, and then sampled to determine fresh weights (FW) of shoot **(A)** and roots **(B)**, Cd concentration in leaves **(C)**, xylem sap **(D)** and roots **(E)** were determined by ICP-MS. Data are mean ± SDs of three independent biological replicates. Bars with the same letters are not significantly different at *P* < 0.05 using the LSD method. Significant differences from ZH11 were determined by Student’s t test (^∗^*P* < 0.05).

Cd tolerance was determined in two randomly selected *CAL2*-overexpression lines and a transgene-negative control, ZH11. Rice seedlings were grown hydroponically for 14 days in a solution supplemented with 0 or 5 μM Cd. The results revealed that the *CAL2*-overexpression transgenic lines, M306-8 and M306-10, had similar fresh weights as the wild-type ZH11 under 0 or 5 μM Cd treatment conditions (Figures 7A,B). Results of our previous studies suggested that the defensin-like protein, CAL1, mediates the efflux and partitioning of Cd in rice (Luo et al., 2018), but whether this protein can regulate Cd tolerance remains unknown. A Cd tolerance assay also performed using *cal1* mutants and the wild-type control, TN1; the *cal1* mutants were generated by CRISPER/Cas9 as reported by Luo et al. (2018). The results suggest that the *cal1* mutants have similar fresh weights of shoots and roots as the wild-type TN1 under 0 or 5 μM Cd treatments (Supplementary Figure S5). Compared with that in the transgenic negative control, ZH11, the concentration of Cd in leaves and xylem sap significantly increased in the *CAL2*-overexpression lines (Figures 7C,D); however, there was no significant differences in root Cd content between the *CAL2*-overexpression lines and the transgenic negative control, ZH11 (Figure 7E). These results indicate that CAL2 positively regulates Cd accumulation, but has no effect on Cd tolerance in rice.

## Discussion

In this study, we functionally identified CAL2, the closest homolog of CAL1, which belongs to the plant defensins and mediates Cd accumulation in rice and *Arabidopsis*. CAL2 has Cd binding activity and is localized in the cell wall similar to CAL1 (Figures 3, 4). Our results indicate that CAL2 probably regulates Cd accumulation via a mechanism similar to that used by CAL1 in ectopically overexpression rice and *Arabidopsis* lines. CAL1 is expressed preferentially in the root exodermis and xylem parenchyma cells. It is involved in Cd chelation and secretion from the cytosol to the extracellular spaces, and consequently it lowers the cytosolic Cd concentration and drive long-distance Cd transport via the xylem vessels (Luo et al., 2018).

*CAL1* specifically regulated Cd allocation to rice leaves and straw, and had no effect on Cd accumulation in rice grains. However, overexpression of *CAL2* positively regulated Cd accumulation in the leaves, straw, and grains of rice. *CAL1* and *CAL2* show different tissue-specific expression. *CAL1* responded to Cd stress at the mRNA level, but *CAL2* did not.

A previous review article suggested that the small peptide expressed in the root tips of *A. thaliana* regulates the initiation and elongation of lateral roots and the maintenance of root apical meristem through a receptor kinase-signaling pathway (Grienenberger and Fletcher, 2015). CAL2 is mainly expressed in the root tips and may act as a peptide signal to control the growth and development of plant roots. However, when plants suffer from Cd stress, *CAL2* may also regulate gene expression for the transport of Cd to adapt to this stress. This requires further studies using genetic assays to fully understand the mechanisms involved.

In our opinion, CAL2 possesses Cd chelation activity, and may enhance Cd tolerance when heterologously expressed in *Arabidopsis*; however, our experimental data in this study (Supplementary Figure S3A) suggest that *Arabidopsis* plants heterologously expressing *CAL2* exhibit a Cd-sensitive phenotype. This is an unexpected phenomenon, which might be due to the side effects caused by heterologous overexpression of the *CAL2* gene driven by the *35S* promoter, high Cd accumulation in shoots of *CAL2*-overexpression lines (Figure 4A), or Cd/Na stress mediating nitrate allocation to roots, which promotes stress tolerance (Li et al., 2010; Zhang et al., 2014, 2018). Our results indicate that CAL2 may negatively regulate the root/shoot nitrate ratio under Cd stress conditions to decrease Cd tolerance in *Arabidopsis* (Supplementary Figure S3). We speculate that in ectopically overexpression plants, CAL2 may act as a signal molecule to regulate the expression levels of genes involved in the long-distance transport of nitrate, namely *NRT1.5* and *NRT1.8*, which affect the distribution of nitrate between shoots and roots, and thus, affect the tolerance of plants to Cd. *CAL2* specifically regulates Cd accumulation (Figure 6), but has no effect on Cd tolerance in rice. This provides a new molecular tool for controlling Cd accumulation in rice and could be used for phytoremediation in other crop species.

## Conclusion

In this study, we functionally characterized the defensin protein, CAL2, and determined its contribution to Cd accumulation. CAL2 shares 66% identity with CAL1, is expressed mainly in roots, and is unaffected by Cd stress. The promoter-*GUS* assay showed that *CAL2* is expressed in root tips. The results of transient expression of the CAL2-mRFP fusion protein indicated that CAL2 is localized to the cell wall. The *in vitro* Cd binding experiment revealed that CAL2 have Cd chelation activity. Our findings indicate that CAL2 positively regulates Cd accumulation in ectopically overexpression lines of rice and *Arabidopsis*, and could play a potential role in phytoremediation in other species.

## Data Availability Statement

All datasets generated for this study are included in the article/Supplementary Material.

## Author Contributions

J-SL and ZZ designed the experiments and analyzed the data. J-SL performed most of the experiments. J-SL, AI, and ZZ wrote the manuscript. All authors contributed to the initial design of the project, and read and approved the manuscript.

## Conflict of Interest

The authors declare that the research was conducted in the absence of any commercial or financial relationships that could be construed as a potential conflict of interest.

## Abbreviations

*CAL1*: cadmium accumulation in leaf 1
*HD5*: human defensin 5
ICP-MS: inductively coupled plasma mass spectrometry
*mRFP*: monomer red fluorescent protein gene
*PDF2s*: defensin type 2 genes
RT-qPCR: quantitative reverse transcription polymerase chain reaction.
