# Supplementary material for: Overexpression of a Defensin-Like Gene CAL2 Enhances Cadmium Accumulation in Plants
Source: Front Plant Sci. 2020 Feb 27;11:217. doi: 10.3389/fpls.2020.00217 (PMC7057248; doi:10.3389/fpls.2020.00217)

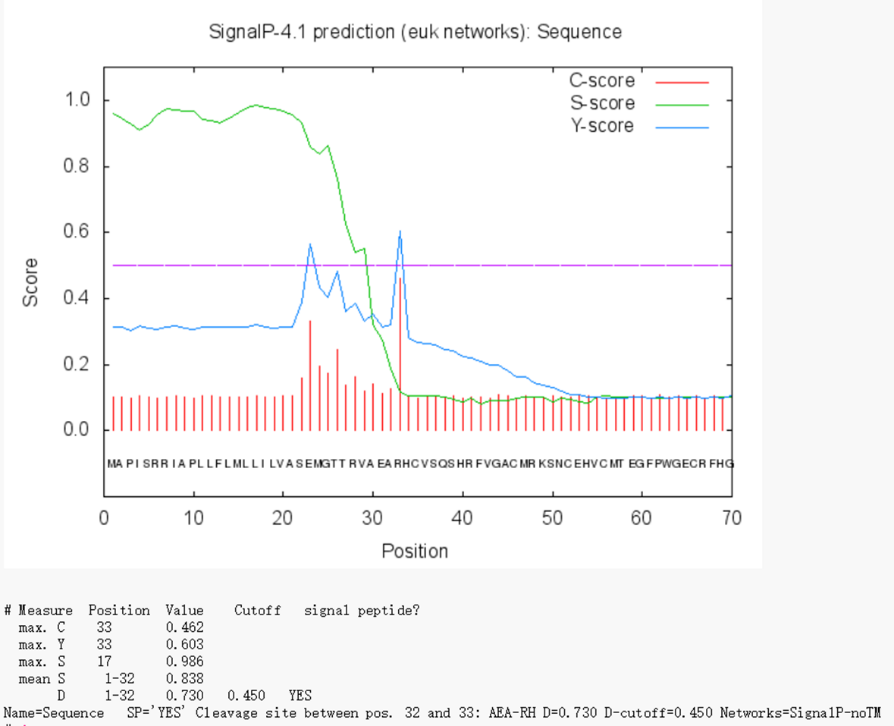


Supplementary Figure S1. CAL2 secretion signal peptide predication by web tool SignaIP 4.0 server. The signal peptide prediction cleavage site is between position 32 and 33 amino acids.


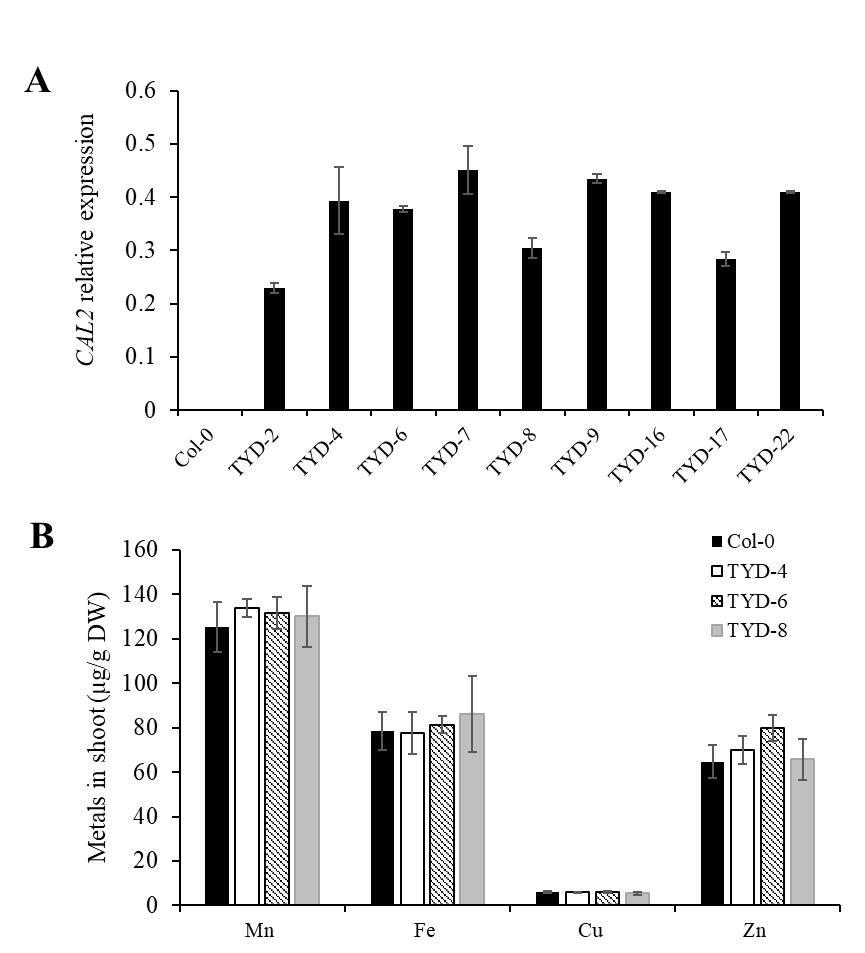


Supplementary Figure S2. Metal accumulation assay of *CAL2* homozygous overexpression lines. Four weeks old of *35S*::*CAL2-mRFP* transgenic Arabidopsis plants and Col-0 were exposed to 10 μM Cd for 3 days. (A) The plants root was then sampled to determine expression levels of *CAL2*, *Actin2* was used as an internal control. (B) Metals concertation in shoots was determined by ICP-MS. Data are mean ± SD of three biological replicates.


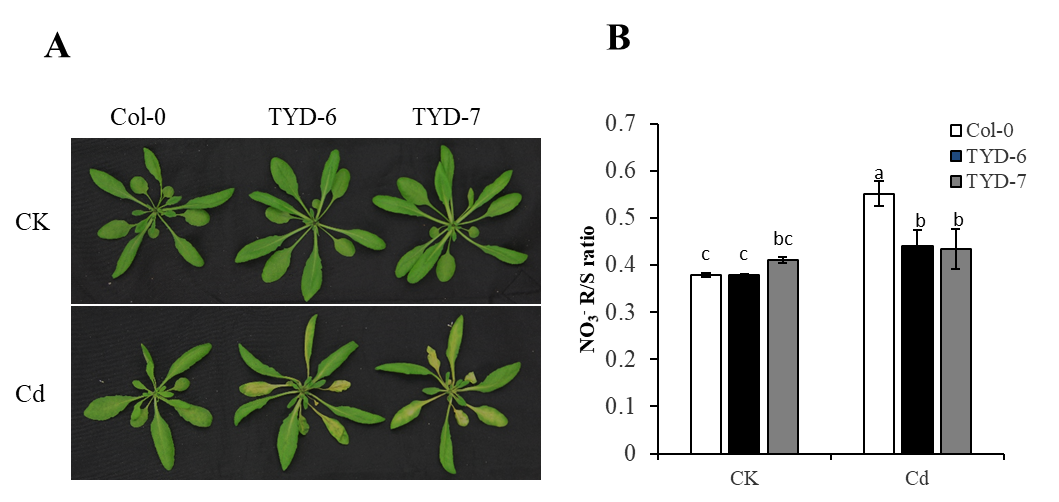


Supplementary Figure S3. Ectopic expression of *CAL2* enhanced Cd sensitively in Arabidopsis.

Four weeks old of hydroponic grown *35S*:: *CAL2-mRFP* transgenic plants were exposed to 20 μM Cd for 3 days. (A) Heterologous overexpression of *CAL2* enhanced Cd sensitivity in Arabidopsis. (B) Heterologous overexpression of *CAL2* decreased root/shoot nitrate ratio in Arabidopsis. Data are mean ± SD of three biological replicates. Bars with the same letter are not significantly different at *P* < 0.05 using the LSD method.


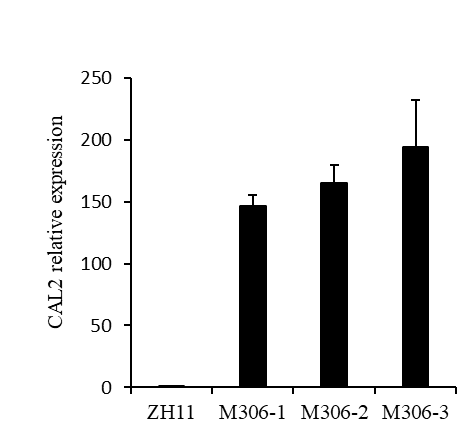


Supplementary Figure S4. Identification of *CAL2* overexpressed rice. ZH11 and *35S*::*CAL2-mRFP* transgenic plants were grown in heavy metal polluted. The plants leaf was then sampled to determine expression levels of *CAL2*, Data are mean ± SD of three biological replicates. *Actin1* was used as an internal control.


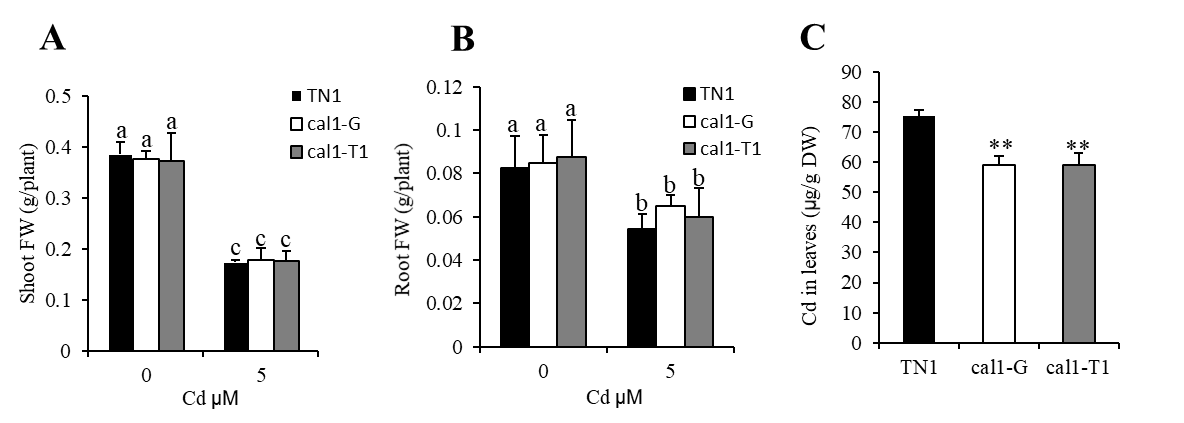


Supplementary Figure S5. Cd tolerance assays of *cal1* mutant plants

Transgene‐negative control TN1 and *cal1* mutant rice seedlings were grown for 14 days in hydroponics solution supplemented with 0 or 5 µM Cd, and then sampled to determine fresh weights (FW) of shoot (A) and roots (B), Cd concertation in leaf (C). Data are mean ± SD of three biological replicates. Bars with the same letter are not significantly different at *P* < 0.05 using the LSD method. Significant differences were determined by Student's t test (***P* <0 .01)


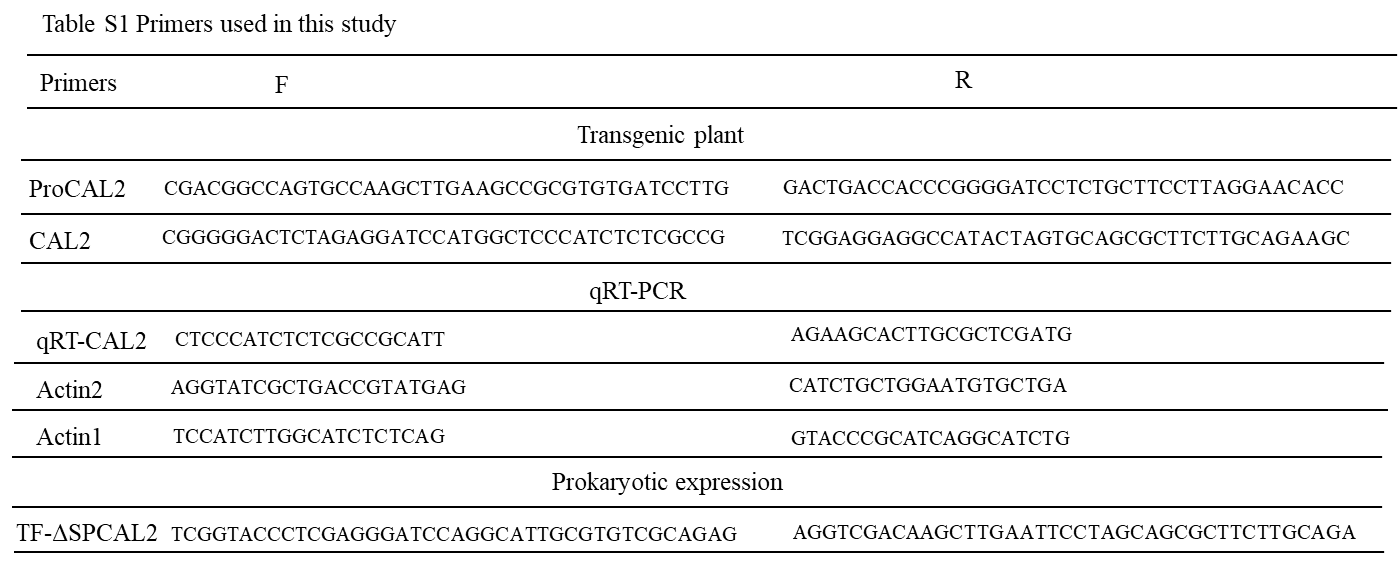

Supplement: Supplementary file 1 [file Data_Sheet_1.docx]
